# Perovskite solar cells in N-I-P structure with four slot-die-coated layers

**DOI:** 10.1098/rsos.172158

**Published:** 2018-05-16

**Authors:** Daniel Burkitt, Justin Searle, Trystan Watson

**Affiliations:** SPECIFIC, Swansea University, Bay Campus, Fabian Way, Crymlyn Burrows, Swansea SA1 8EN, Wales, UK

**Keywords:** perovskite, slot-die, scale-up, N-I-P

## Abstract

The fabrication of perovskite solar cells in an N-I-P structure with compact titanium dioxide blocking, mesoporous titanium dioxide scaffold, single-step perovskite and hole-transport layers deposited using the slot-die coating technique is reported. Devices on fluorine-doped tin oxide-coated glass substrates with evaporated gold top contacts and four slot-die-coated layers are demonstrated, and best cells reach stabilized power conversion efficiencies of 7%. This work demonstrates the suitability of slot-die coating for the production of layers within this perovskite solar cell stack and the potential to transfer to large area and roll-to-roll manufacturing processes.

## 1. Introduction

Solution-processable materials for photovoltaics are subjects of a great deal of research and commercial development due to the potential for high-throughput manufacture and so lower production costs than many conventional photovoltaic manufacturing techniques [1]. Of recent interest are organic–inorganic alkylammonium lead halide perovskite materials that offer the potential for lower materials costs, high efficiencies and solution processing [2–4]. Many deposition techniques for solution-processed solar cells have been proposed, these include printing techniques such as screen [5,6], ink-jet [7] and gravure [8] printing and coating techniques such as spin, spray [9–11], bar [12] and slot-die coating [13–20]. Of these techniques, slot-die coating is of particular interest as it can be used to deposit solutions with a wide range of rheological properties on to both rigid or flexible substrates, allows for simple patterning of materials in stripes or blocks and as a pre-metered coating method results in little materials wastage during coatings. It also has the potential for high coating line speeds and roll-to-roll production [21,22], all helping to give a real possibility of high-throughput, low-cost manufacture.

A number of studies have already demonstrated the slot-die coating of a perovskite layer for use in a photovoltaic device stack. Many of these have also demonstrated the deposition of a number of the other layers in a range of different device stacks on rigid and flexible substrates. For N-I-P-type devices, these include zinc oxide and phenyl-C_61_-butyric acid methyl ester (PCBM) electron collection layer [13,14] and spiro-MeOTAD and poly-3-hexylthiophene (P3HT) as hole-transport layer [13,14,18]. A commonly used N-I-P perovskite solar cell device stack is one using a mesoporous titanium dioxide scaffold. The slot-die coating of the perovskite layer in this stack has previously been demonstrated [17], but slot-die coating of the other layers in the stack, has not, in particular the titanium dioxide blocking layer where a relatively thin and very uniform layer is required and poses considerable coating challenges.

**Figure 1.**
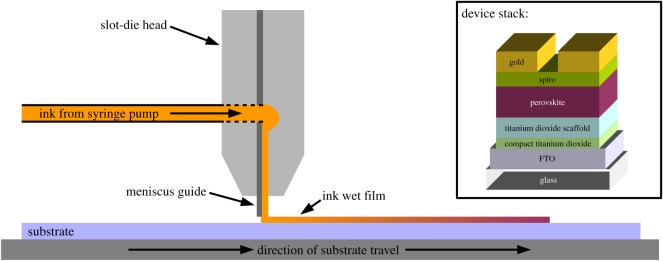
A schematic of the slot-die coating process used and (inset) the device stack.

In this work, the deposition of four layers of a perovskite solar cell made on a rigid fluorine-doped tin oxide (FTO)-coated glass substrate using slot-die coating is demonstrated. A device stack using an N-I-P structure is shown with compact titanium dioxide blocking layer, mesoporous titanium dioxide scaffold layer, single-step methyl-ammonium lead iodo chloride perovskite layer and spiro-MeOTAD hole-transport layer, all slot-die coated followed by an evaporated gold top contact. A schematic of the slot-die coating set-up and the device stack are shown in figure 1. The slot-die-coated layers are compared to ones made using conventional spin or spray coating methods. The deposition method used for layers strongly determines the formation and morphology of these and how they stack and the resulting interfaces that form, as well as the type and number of defects that form. For instance, the size of grains and number of grain boundaries in the perovskite film can influence the number of electronic trap states that form and the resulting performance of the device. As well as this, any pin-holes or other defects through the film can allow the formation of unfavourable interfaces between the opposite charge selective layers or electrode materials and could result in shunt losses or short circuits [23]. In particular for the titanium dioxide blocking layer, cracks or pin-hole defects can result in trap states that increase photogenerated charge recombination and leave exposed areas of the underlying electrode that can provide a pathway for shunt leakage through poorly aligned energy levels of the interfacing materials [24]. The formulation of the inks is critical to achieve good slot-die coating quality and to help avoid the use of toxic and unfavourable materials as well as achieving good performance.

## 2. Experimental

FTO-coated glass (TEC8) substrates were cleaned sequentially with acetone, dilute Hellmanex, water and 2-propanol. A bench-top slot-die coating unit, with in-line forced hot air oven, positioned in a fume-hood in a clean-room environment with controlled humidity (30% RH), was used for coating. The slot-die coating head had a coating width up to 100 mm, patterned shims were used to define coated areas and corresponding meniscus guides were used with the slot-die head to help form the meniscus between the slot-die head lips and substrate [25], the length of the meniscus guide protrusions was 1000 μm. The gap height between the meniscus guide protrusions and substrate was set using feeler gauges and micrometer adjustment screws and set to as close to 50 μm as possible for each coating, resulting in an approximately 1050 μm gap between the slot-die lips and substrate.

Compact titanium dioxide blocking layer formulations were made up by dilution of a solution of titanium diisopropoxide bis(acetylacetonate) (Sigma-Aldrich) in 2-propanol with various alcohols depending on coating method, for spray coating 2-propanol was used, for spin coating 1-butanol [3] and for slot-die coating a range of alcohols were assessed and methanol was chosen for its good blocking layer quality. Slot-die-coated layers used a coating speed of 0.1 m min^−1^, a coating width of 50 mm, a pump rate of 0.05 ml min^−1^ giving a wet film thickness of approximately 10 μm. The formulations were prepared then filtered using 0.2 μm pore size polytetrafluoroethylene syringe filters and then used within a few hours. Spin-coated layers were dried at 140°C for 10 min on a hotplate and slot-die layers were passed through the coater ovens at 105°C for 3 min, to remove residual solvent before coating of the mesoporous titanium dioxide layer.

The mesoporous titanium dioxide layer was deposited using spin or slot-die coating, formulations were made up by dilution of DSL-18NRT titanium dioxide paste (Dyesol). A range of alcohols were trialled for the slot-die coating formulation, these being methanol, ethanol, 2-propanol, 1-propanol, 1-butanol and cyclohexanol, and the viscosity and surface tension measured as well as the contact angle on compact titanium dioxide blocking layer. 1-butanol was found to give the best coating quality, assessed by film image analysis, formulations with a solid content of 0.5 wt% were used to make devices, and for spin coating ethanol was used in a 2 : 7 wt% ratio. For films for image analysis a small amount of dye (pararosaniline acetate) was added to give the films colour, and films were scanned with a digital flat-bed scanner without first sintering. The formulations were stirred constantly to avoid agglomerations and filtered using 0.45 μm pore size regenerated cellulose syringe filters directly before use. The slot-die-coated layer was deposited at a coating speed of 0.1 m min^−1^ and a pump rate of 0.05 ml min^−1^ giving an approximately 10 μm wet film thickness and initially dried by passing through the coater ovens at 105°C (3 min). The mesoporous layers were all further dried at 140°C (10 min) to remove residual solvent then 325°C (10 min) to burn off organic materials followed by a 550°C sinter for 30 min.

The perovskite layer was deposited by spin or slot-die coating from a formulation of lead chloride and methyl ammonium iodide in a 1 : 3 molar ratio in dimethylformamide, for spin coating a 40 wt% solution was used and for slot-die coating 24 wt%, both depositions were carried out in air in a humidity-controlled clean-room (30% RH). The spin-coated solution was dispensed onto substrates (50 by 50 mm), spun at 2000 r.p.m. for 45 s and then the substrates were placed on a hot-plate at 90°C for 3 min before being further dried at 100°C in a fan oven for 90 min, to induce crystallization of the perovskite [26]. The slot-die-coated films were coated at 1.0 m min^−1^ with a pump rate of 0.5 ml min^−1^. After coating, the films were then paused on the coating line for 2 min before directly passing on through the coating machine’s in-line oven at approximately 105°C at a conveyor speed of 0.1 m min^−1^ giving an oven residence time of 3 min. The films were then further dried in a fan oven in air for a further 90 min at 100°C.

The hole-transport material was spiro-MeOTAD. For spin-coated samples, spiro-MeOTAD (75 mg ml^−1^ in chlorobenzene, with 10 μl 4-tert-butylpyridine [27] and 20 μl 600 millimolar bis (trifluoromethane)sulfonimide lithium salt [28] in acetonitrile per millilitre of solution) was coated at 2000 r.p.m. for 45 s in a nitrogen-filled glove-box. For slot-die coating, to avoid the use of highly toxic chlorobenzene, a formulation the same as for spin coating was prepared but with toluene instead of chlorobenzene as solvent, this was then further diluted with more toluene by one part to two parts toluene. This was deposited in air at a coating speed of 1.0 m min^−1^ and a pump rate of 0.5 ml min^−1^ and allowed to dry at room temperature through the bench-top coater ovens. Films were left overnight (16 h) in a box with an atmosphere of air, with desiccant to reduce the humidity, to promote oxidation of the films.

Top contacts were prepared by thermal evaporation, under vacuum, of gold using a shadow mask to define pixel areas. Cells were masked to 0.09 cm^2^ for photovoltaic testing.

Current–voltage testing of devices was performed using a class AAA solar simulator (Newport Oriel Sol3A) as light source calibrated to one AM1.5 sun equivalent intensity using a reference cell fitted with a KG5 filter (Newport Oriel 91150-KG5). Current–voltage curves, in reverse and forward scan directions, were collected using a Keithley 2400 source measure unit between bias of 1.1 and −0.1 *V* at a scan rate of 0.15 V s^−1^; 5 s of light soaking was applied to the cell before scanning. Stabilized power output measurements were made by illuminating the cell under the solar simulator and holding the cell at the maximum power voltage, found from the reverse and forward current–voltage scans, while measuring the development of current over time, typically 60 seconds.

Cyclic voltammetry measurements were made in a three-electrode set-up using a platinum counter electrode, a calomel reference electrode and the substrate with a defined exposed area as a working electrode. An electrolyte solution of potassium ferri/ferrocyanide was made up to 0.5 millimolar of both potassium ferricyanide and potassium ferrocyanide in a 0.5 molar potassium chloride aqueous solution. Scans were taken using a potentiostat taking cyclic scans from −0.15 to 0.6 V with a scan rate of 100 mV s^−1^.

Scanning electron microscopy (SEM) images were collected on Jeol JSM-7800F field emission gun electron microscope. X-ray diffraction (XRD) spectra were collected using a Bruker D8 Discover instrument with a CuK*α* beam at 40 kV and 40 mA.

## 3. Results and discussion

### 3.1. Blocking layer

The conventional method for depositing a compact titanium dioxide blocking layer is spray pyrolysis, where a precursor solution is spray-coated onto the substrate, which is held at an elevated temperature, usually above 300°C, and then sintered at a temperature above 450°C. Spin coating can also be used to produce blocking layers, generally using a room-temperature spinning stage followed by heating and sintering steps. An ideal blocking layer will form a uniform conformal coating over the surface of the FTO substrate and be free from pin-holes, cracks and other defects that would expose the underlying FTO surface. Spray pyrolysis results in a very conformal coating and generally high-quality blocking layers, but the high temperature used for the coating stage is not attractive for a manufacturing process, due to the added energy costs. The spin coating process is generally performed at room temperature, so is more attractive, but usually results in less-conformal and less-uniform coatings and lower-performance blocking layers. Despite this, spin-coated layers have been shown to be adequate for use in high-performance perovskite solar cells [24].

To achieve the best possible blocking layer quality through a slot-die-coated film a range of inks with various rheologies were developed. The inks were formulated by dilution of a solution of titanium diisopropoxide bis(acetylacetonate) in IPA with other alcohols to reduce the concentration to a level suited for slot-die coating and alter the rheology and blocking layer film formation. The alcohols studied were methanol, ethanol, IPA, 1-propanol, 1-butanol and cyclohexanol, inks were formulated using these solvents and coatings of each made.

Formulations were prepared using each of the inks and slot-die meniscus guide coatings made, the coating parameters were kept constant for each ink and all were sintered at 550°C to form a crystalline material. The transparent nature of the thin films makes optical inspection of the film coating quality difficult. To assess the quality of the compact titanium dioxide blocking layer, cyclic voltammetry was used, adapting a method given by Kavan *et al.* [29]. A redox couple of potassium ferri/ferrocyanide was used as this would be made up of small enough molecules to penetrate small pin-holes and cracks in the titanium dioxide blocking layer and reach the FTO electrode. An estimation of the surface coverage of the blocking layer over the electrode can be found by looking at the magnitude of the anodic current density of the cyclic voltammogram, as the titanium dioxide should form a dielectric contact with the redox couple and so should not result in any anodic current, so all anodic current should only result from exposed FTO [30,31]. This can then be compared to a voltammogram of a bare FTO electrode and the relative anodic current density used to determine an approximate surface coverage. The use of the small redox couple should give an idea of the worst-case scenario of hole blocking ability, as in a device to form a shunt pathway between the hole-transport material and FTO; the HTM would have to penetrate through the other layers of the device and then through the pin-holes or cracks of the blocking layer and as the HTM materials used here are larger than the redox couple, they should not be able to do this to the same extent. This does not account for other shunt pathways between the perovskite layer or with the scaffold layer or any blocking ability resulting from the perovskite layer itself, which could also impact device performance.

**Figure 2.**
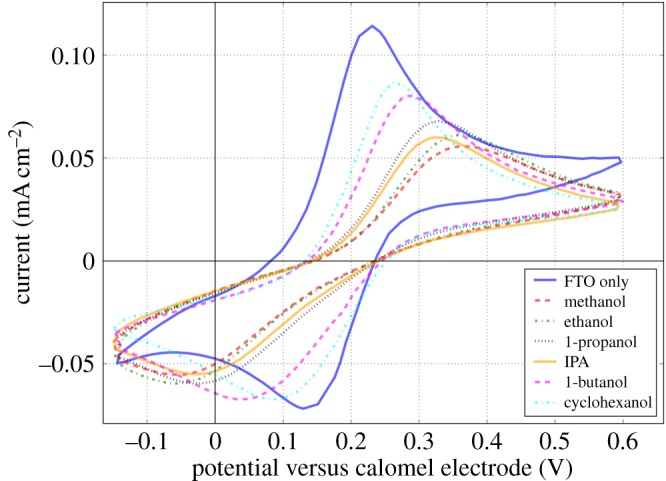
Cyclic voltammograms of FTO substrates coated with compact titanium dioxide layers deposited by slot-die coating from inks based on dilution using various alcohols.

Cyclic voltammograms of the blocking layers prepared from each ink are given in figure 2, clearly the lowest anodic current compared to bare FTO is found for the methanol-based film with the higher alcohols giving progressively greater anodic currents. This suggests that the methanol-based formulation results in the greatest surface coverage of titanium dioxide over the FTO electrode and should result in better device performance.

**Figure 3.**
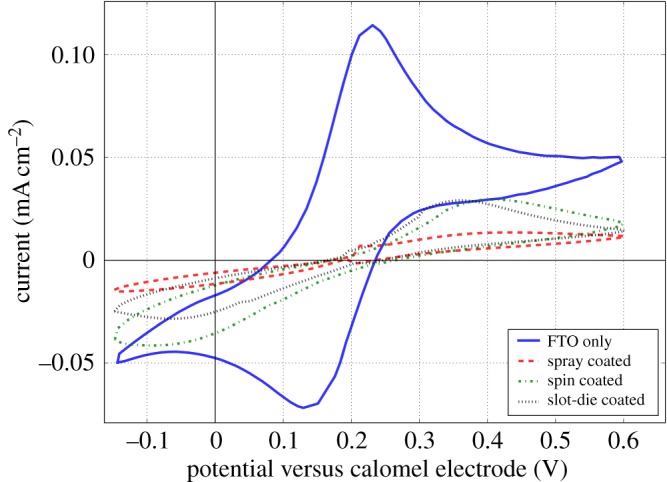
Cyclic voltammograms of FTO substrates coated with compact titanium dioxide layers deposited by spray, spin or optimized slot-die coating.

A further improvement was made to the slot-die-coated blocking layer by first coating a methanol-based formulation with a solid content of 6.25 wt%, drying in the coater ovens for 3 min, followed by coating with a lower solid content ink of 3.125 wt%, followed by further drying. The spin coating process [3,32] is based on the coating of multiple layers of titanium dioxide precursor, and an improvement in device performance has also been observed for multi-coated screen-printed blocking layers [33], as is the case here. Films made using this method were further analysed using cyclic voltammetry and used to make complete devices. Comparison blocking layers were also prepared by spray or spin coating, cyclic voltammograms for all the films are shown in figure 3, for spray-coated blocking layer the anodic current is reduced greatly compared to bare FTO, which is similar to that found elsewhere [24,29]. It is evident that both the spin- and slot-die-coated blocking layers show greater anodic current densities than the spray-coated layers, so indicating worse surface coverage of the FTO electrode. The magnitude of the anodic current is similar to that found elsewhere for spin-coated blocking layers, and the slot-die-coated layer’s anodic current is similar to that of the spin coated layer. The spray-coated layer results in an estimated surface coverage of 88% and for the spin- and slot-die-coated 74% and 76%, respectively. Although the slot-die-coated surface coverage is not as high as the spray-coated layer it is still promising, as high-quality devices can still be made using the spin-coated blocking layer [3,24,30] that shows similar levels of coverage.

To further investigate if the slot-die-coated blocking layer would be adequate, photovoltaic devices were fabricated and compared to ones using a spray- or spin-coated layer. The JV parameters calculated for the devices are given in table 1; the devices made using slot-die coating (structure: C) show a small increase in open circuit voltage (Voc) compared to spray (structure: A) blocking layer but similar to spin coated (structure: B). Both slot-die coated and spin coated show a reduction in short-circuit current density (Jsc) and fill factor (FF) compared to spray coated, which leads to a reduction in the median power conversion efficiency (PCE). This suggests the hole blocking ability of the spin- and slot-die-coated films are similar and lower than the spray coated, as seen in the cyclic voltammetry measurements, which results in increased shunt losses. This is confirmed by the values of shunt and series resistance found for the different blocking layer deposition method devices. All deposition methods result in devices with similar reverse scan light series resistances, calculated from the gradient of the JV curves around Voc, with median values of 3.27, 3.11 and 3.31 Ω cm^2^ for the spray, spin and slot-die coated, respectively. However, there is a reduction in the reverse scan light shunt resistance for both the spin- and slot-die-coated blocking layers compared to the spray coated from 630 to 344 and 470 Ω cm^2^ for the spray, spin and slot-die coated, respectively, which follows the trend in PCEs.

**Table 1.** Device structures, layer deposition methods and median reverse JV scan device performance

structure	blocking layer	scaffold	perovskite	hole-transport material	Voc	Jsc	FF	PCE
A	spray	spin	spin	spin	0.76	20.89	67.27	10.39
B	spin	spin	spin	spin	0.78	18.53	60.40	7.97
C	slot-die	spin	spin	spin	0.78	19.16	64.24	9.39
D	slot-die	slot-die	spin	spin	0.83	15.07	66.80	8.21
E	slot-die	slot-die	slot-die	spin	0.89	18.33	72.68	11.74
F	slot-die	slot-die	slot-die	slot-die	0.80	15.76	45.95	5.73

### 3.2. Titanium dioxide scaffold

The titanium dioxide scaffold layer for perovskite solar cells is usually deposited by screen [34], spin [32], doctor blade [3] or bar coating [17] from an ink and then further dried and sintered. To achieve the best performance, the layer must have the appropriate porosity and have sufficient necking to achieve good charge transport, as well as being uniform and free from defects such as pin-holes and mesh markings that could otherwise increase the shunt losses within the device. The titanium dioxide inks used here for spin and slot-die coating were formulated from a commercial screen printing paste with high viscosity developed for use in dye-sensitized solar cells that has also shown good performance when used in perovskite solar cells [2,11,35]. For the titanium dioxide scaffold layer formulations, a range of alcohols were investigated as solvent for dilution of the viscous starting paste to achieve the best rheology for slot-die coating. The alcohols chosen significantly change the rheology of the ink in various ways, particularly in terms of the viscosity and wetting, both important factors in achieving good slot-die coating. The viscosity and surface tension of the inks and the initial contact angle formed by the inks on a compact titanium dioxide surface are given in table 2 for each ink trialled.

**Table 2.** Rheology of mesoporous titanium dioxide inks.

	surface tension	viscosity	contact angle
scaffold ink solvent	(mN m^−1^)	(mPa s)	(°)
methanol	19.35	2.84	14.57
ethanol	17.75	5.52	19.62
IPA	18.90	9.34	21.25
1-propanol	20.13	9.74	24.24
1-butanol	22.89	13.32	27.18
cyclohexanol	23.47	143.03	66.58

**Figure 4.**
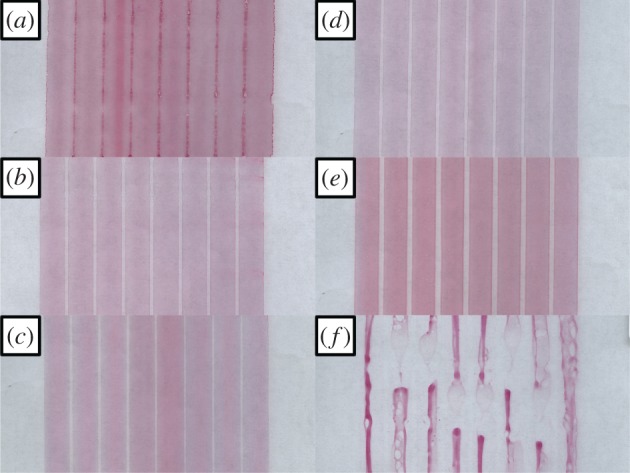
Optical images of sections, 10 cm by 5cm, of titanium dioxide scaffold layer with added dye meniscus guide stripe coated from various solvents, methanol (*a*), ethanol (*b*), IPA (*c*), 1-propanol (*d*), 1-butanol (*e*), cyclohexanol (*f*).

Films were slot-die meniscus guide-coated using a striped pattern (eight stripes of 7.5 mm width and 1.5 mm gap) to assess the coating quality of each ink. Due to the transparent nature of the coated film, a small amount of dye was added to each ink to enhance the visibility of the films. Digital images of the films produced were taken using a flat-bed scanner, and sections of these are shown in figure 4. The ink based on methanol shows complete gap narrowing of the film with the ink from each stripe wetting into the neighbouring stripes. Methanol also shows the lowest contact angle; its rheology is not suitable for controlled coating and leads to excess wetting of the substrate and so poor film formation. At another extreme, the ink based on cyclohexanol shows a discontinuous film with the coating in each stripe stopping and starting along the coated length; this indicates that a low flow limit is being reached. Using the viscocapillary model [36,37] of slot-die coating, this could be related to the high capillary number (ratio of viscosity and coating speed to surface tension) at this coating speed, due to the high viscosity of the ink, though applying the viscocapillary model to meniscus guide coating is not so well understood. The cyclohexanol ink also shows reticulation and de-wetting across the coated areas which occurred as the ink entered the oven to dry. The other inks show various levels of gap narrowing of the stripes, and uniformity of the edges of the stripes but the 1-butanol ink gave the best overall coating quality and so was used for fabrication of devices.

**Figure 5.**
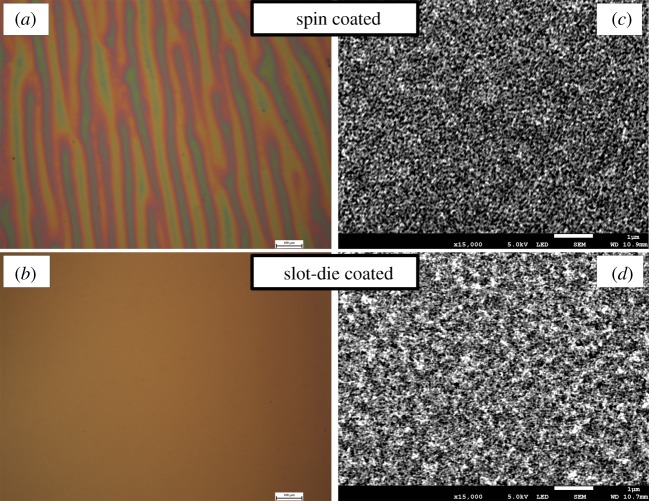
Optical microscope (*a*,*b*) and SEM (*c*,*d*) images of titanium dioxide scaffold layer. Scale bars in the SEM images represent 1 μm and for the optical microscope images 100 μm.

Figure 5 shows optical microscope and SEM images of mesoporous titanium dioxide scaffold layers, on FTO-coated glass substrates with blocking layer, deposited using either spin or slot-die coating. In the SEM images over small areas, the films from both coating methods look very similar with a porous structure with small pores less than 100 nm in diameter visible. But on the larger area optical microscope images, the spin-coated sample shows striation defects [38] with a rough undulating surface, whereas the slot-die-coated films are uniform and planar across the area. This indicates that slot-die coating is suitable for the production of mesoporous titanium dioxide films and can improve on the coating quality compared to spin coating.

Devices were made incorporating the slot-die-coated scaffold layer (structure: D) and compared to the spin-coated scaffold layer (structure: C), see table 1. The slot-die-coated scaffold layer gives slightly worse performance compared to the spin coated, mainly due to a decrease in Jsc which can be attributed to worse perovskite film coverage, seen in SEM images, achieved by spin coating on the more planar slot-die-coated scaffold surface.

### 3.3. Perovskite

Having successfully deposited the blocking and scaffold layers, these were used to prepare devices with the perovskite layer also deposited using slot-die coating and compared to ones with the perovskite layer spin coated. The commonly used methyl-ammonium lead triiodide was selected as the perovskite material to deposit, this was deposited from a mixed halide formulation made from lead chloride and methyl-ammonium iodide, as this has previously shown good performance when slot-die coated and used in the same device stack [17].

A room-temperature coating process was developed to achieve good perovskite crystallization and film coverage, rather than a formulation with a high 40 wt% solid content, as used in spin coating or previously reported slot-die coating [17]. A lower solids content formulation of 24 wt% was used to slow the drying of the film and the nucleation of crystallites within it. To avoid uneven nucleation within the initial wet film caused by thermal gradients established on entering the forced hot air ovens, which would result in poor morphology, once coated the films were then paused on the coating line for 2 min to allow solvent to evaporate at room temperature and generate a uniform initial nucleation within the film before passing on to the oven units where excess solvent was removed, the films were then moved to a fan oven to further dry and crystallize due to the long drying times required and short length of the in-line oven.

**Figure 6.**
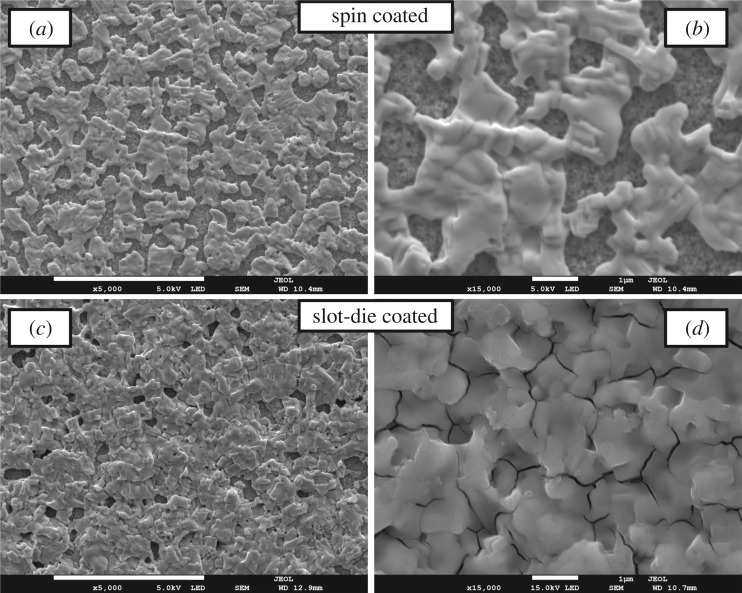
SEM images of spin-coated (*a*,*b*) and slot-die-coated (*c*,*d*) perovskite layers. Scale bars in the images represent 10 μm (*a*,*c*) and 1 μm (*b*,*d*).

Figure 6 shows SEM images of the perovskite layer produced using spin or slot-die coating with the optimized coating conditions. In both cases, gaps of uncoated scaffold layer are visible between the perovskite crystal formations. The perovskite crystals are of the order of a few micrometres in diameter and voids between them and at grain boundaries can be seen. Comparing the surface coverage between the spin- and slot-die-coated films it can be seen that spin-coated films have worse film coverage, showing many voids between the perovskite capping layer formations. The spin-coated films were prepared in air to allow for easier comparison to the slot-die-coated films, rather than in a glove-box environment where better surface coverage is generally achieved. The slot-die-coated films show better surface coverage and a more complete capping layer than the spin coated, but voids and pin-holes are still present.

**Figure 7.**
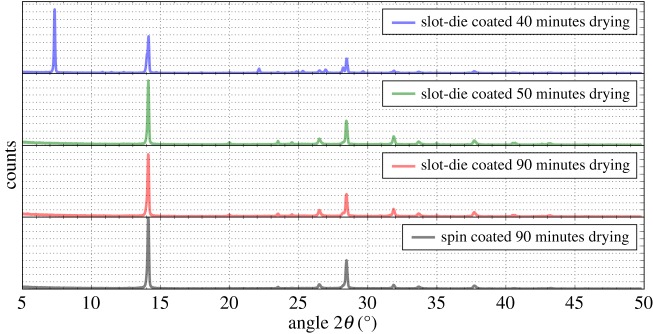
XRD spectra of perovskite films deposited by spin or slot-die coating after various drying times.

The XRD spectra of the perovskite films produced using spin and slot-die coating followed by drying in air are shown in figure 7. In both cases, at 90 min, the peak at a 2*θ* angle of 14.1° is dominant, which is assigned to the 110 reflection of the tetragonal phase, no lead iodide peak, around 12.6°, was found for either spin- or slot-die-coated films indicating good conversion to the final perovskite and no degradation of the material due to excessive heating. To determine the optimal drying time of the slot-die-coated perovskite samples, where the wet film thickness immediately after coating will be substantially greater than for spin-coated films [39], films were coated and dried for a range of times and XRD spectra taken. It was found that to remove all residual solvent and achieve full conversion to the final perovskite a drying time of greater than 50 min was required, indicated by the loss of the low angle peak from the spectra at near 7°, which is attributed to a solvent containing phase [40].

JV results for devices made using the spin-coated (structure: D) or slot-die-coated (structure: E) perovskite layers are given in table 1. The performance for devices with slot-die-coated perovskite layer is very good, with median PCEs of 11.74%, the improvement over the devices with spin-coated perovskite layer is due to an improvement in the Voc to 0.89 V and in the FF to 72.68%. This can be attributed to the slot-die-coated layer achieving a more complete capping layer over the scaffold layer than was possible by spin coating, as seen in SEM images, which is also indicated by the improved reverse scan light shunt resistance of 1108 Ω cm^2^.

### 3.4. Hole-transport material

The final layer slot-die coated was the hole-transport material, spiro-MeOTAD. Slot-die coating of the layer has been shown to give good performance in a similar device structure [18]. For spin-coated films, chlorobenzene is typically used as the solvent for spiro-MeOTAD. For slot-die coating, to avoid using highly toxic chlorobenzene (workplace exposure limit (WEL) time weighted average (TWA): 5 ppm) as the solvent and to find a material more suited to use in a production environment, a lower toxicity solvent toluene (WEL TWA 50 ppm) was used for slot-die spiro-MeOTAD formulations.

**Figure 8.**
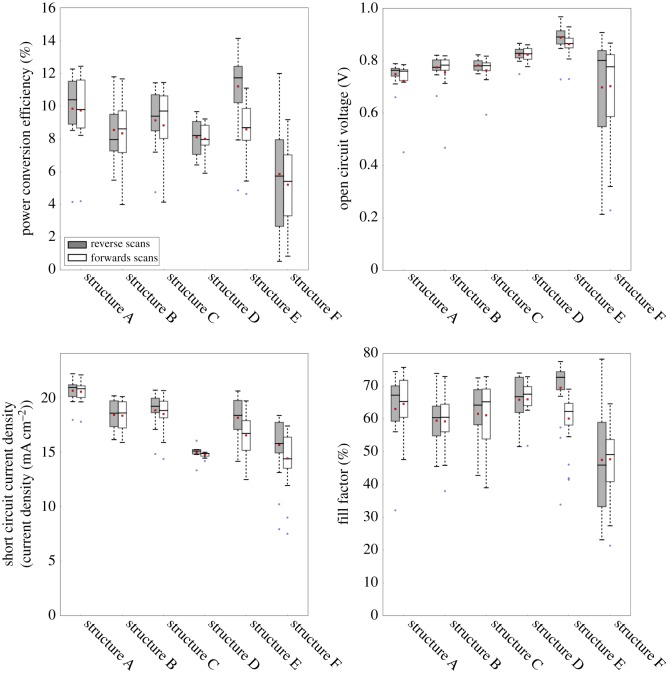
Box-plots of the power conversion efficiencies achieved for devices made using each of the device structures. The boxes represent the first and third quartiles, the horizontal black line the median, the upper whisker the data within 1.5 times the interquartile range of the upper quartile and the lower whisker 1.5 times the interquartile range of the lower quartile, red square the mean and blue dots outliers.

**Figure 9.**
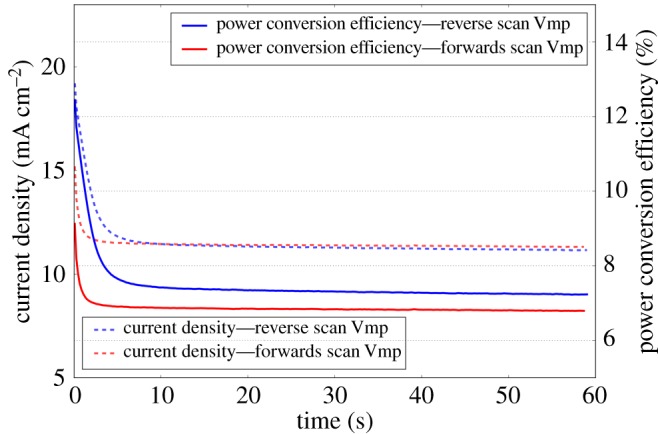
Stabilized current density and power conversion efficiency of fully slot-die-coated hero cell. Cells were held under one sun illumination at the maximum power voltage points found for both the reverse and forward scans and the current allowed to stabilize and the power density and PCE calculated from these. Dashed lines represent the current density and solid lines represent the PCE.

A comparison of the JV performance of devices made with slot-die-coated layers followed by spin-coated spiro-MeOTAD (structure: E) or slot-die coated (structure: F) is given in table 1. The devices with slot-die-coated spiro-MeOTAD layer show a great spread in device performance, as shown in figure 8, with a standard deviation of 3.4 in the PCE data, as well as a low median PCE of 5.73. The devices show a slightly increased series resistance of 5.58 Ω cm^2^ compared to 3.97 Ω cm^2^ for the spin-coated spiro-MeOTAD devices and a much lower reverse scan light shunt resistance of 174 Ω cm^2^. This suggests that the film formation of the spiro-MeOTAD layer is poor and there are pin-holes or de-wetting defects in some areas, resulting in poor performance cells and better film coverage in other areas resulting in higher performance cells. Nonetheless, some cells show high efficiency with a hero PCE of 11.99%, indicating with further improvements to the hole-transport layer homogeneity high average device efficiencies could be achieved. The anomalous hysteresis phenomenon displayed by perovskite solar cells can give rise to apparently erroneous device efficiencies from standard JV curve measurements. One method used to give a more reliable estimate of device efficiency is the stabilized current measurement [41], where the device is held at the maximum power point voltage of the JV scan and the power output recorded over time. Often the power output will change and stabilize and this stabilized power output is used to calculate a ‘stabilized device efficiency’. For the hero cell of structure F a stabilized PCE of 7.01% (average of reverse and forwards scans holding at maximum power voltage point for scan direction) was recorded, as shown in figure 9. The initial rapid drop in measured current density and so PCE [42] is due to the anomalous hysteresis phenomenon. An initial distribution of mobile ions in the perovskite structure, combined with the particular device interfaces and trap state distributions along with the photo-generated charges and bias condition, results in an initially favourable charge collection efficiency, but as a new mobile ion distribution forms under the changed internal field a less favourable equilibrium mobile ion distribution forms, which results in a worse charge collection efficiency and in a lower stabilized device performance [43]. Reducing the hysteresis shown by the devices would be expected to improve the stabilized device performance, which could be achieved by modification of the device interfaces and perovskite, e.g. by more careful control of precursor ratios, improvement of the matching between perovskite and charge collection layer interfaces [44], or reduction in the number of trap states at open grain boundaries by depositing a more compact perovskite film morphology [45] that could be achieved by improvements in the ink formulation and slot-die-coating process.

## 4. Conclusion

This work demonstrates that many of the layers in a perovskite solar cell stack can be easily slot-die coated, including the compact blocking layer. Through the choice of materials and solvents for formulations and optimization of coating conditions reasonable device performance was achieved for four layer slot-die-coated cells. Good quality slot-die-coated compact blocking layers were produced by using a methanol-based ink and two coating steps, one with a higher solids content followed by one with a lower solids content, which resulted in devices with improved performance compared to those with layers produced using spin coating. Improvements to the quality of the scaffold layer were made compared to a spin-coated layer and the ink rheology optimized for the slot-die coating conditions used. Through optimization of the perovskite coating conditions and formulation, layers of perovskite with improved surface coverage compared to spin-coated layers were produced and resulted in devices with good performance. Furthermore the highly toxic chlorobenzene solvent commonly used for the hole-transport layer was replaced with a less toxic alternative.

Many other challenges need to be addressed before the device performance of slot-die-coated cells reaches that of the best spin-coated cells. Of critical importance is achieving complete film coverage of the perovskite layer over the underlying layers. The film coverage and uniformity influences both the current collected and losses due to shunt leakages, and maximizing this will help to improve device performance as well as improving overall device yield. High-temperature steps such as those used to sinter the titanium dioxide layers need to be removed to reduce the cost of the production process and allow for the use of a wider range of substrates such as plastic foils. The long drying step used here for the perovskite layer would also need to be reduced substantially. The quality of the blocking layer would also need to be improved with the surface coverage at least as good as conventional spray coating, but the results presented here show that devices can still be produced with a lower surface coverage. The coating speed and quality of all the layers needs a great deal of optimization to achieve economically viable throughput and yields, which will require much modification of the rheology of formulations to suit slot-die coating better. As well as this, material sets using solvents safe to use on large area coating lines will have to be developed, particularly for the perovskite layer where DMF, as used in this work and many others, would not be suitable without very stringent safety precautions [46,47].

## Data accessibility

Datasets have been deposited in the Dryad Digital Repository http://dx.doi.org/10.5061/dryad.r572v [48].
